# An effective multisource informed consent procedure for research and clinical practice: an observational study of patient understanding and awareness of their roles as research stakeholders in a cancer biobank

**DOI:** 10.1186/1472-6939-14-30

**Published:** 2013-07-30

**Authors:** Silvia Cervo, Jane Rovina, Renato Talamini, Tiziana Perin, Vincenzo Canzonieri, Paolo De Paoli, Agostino Steffan

**Affiliations:** 1 CRO-Biobank, Centro di Riferimento Oncologico, IRCCS, Aviano, Italy; 2 Department of Epidemiology and Biostatistics, Centro di Riferimento Oncologico, IRCCS, Aviano, Italy; 3 Division of Pathology, Centro di Riferimento Oncologico, IRCCS, Aviano, Italy; 4 Scientific Directorate, Centro di Riferimento Oncologico, IRCCS, Aviano, Italy

**Keywords:** Informed consent, Patients, Understanding, Awareness, Biobank, Patient education, Empowerment

## Abstract

**Background:**

Efforts to improve patients’ understanding of their own medical treatments or research in which they are involved are progressing, especially with regard to informed consent procedures. We aimed to design a multisource informed consent procedure that is easily adaptable to both clinical and research applications, and to evaluate its effectiveness in terms of understanding and awareness, even in less educated patients.

**Methods:**

We designed a multisource informed consent procedure for patients’ enrolment in a Cancer Institute Biobank (CRO-Biobank). From October 2009 to July 2011, a total of 550 cancer patients admitted to the Centro di Riferimento Oncologico IRCCS Aviano, who agreed to contribute to its biobank, were consecutively enrolled. Participants were asked to answer a self-administered questionnaire aim at exploring their understanding of biobanks and their needs for information on this topic, before and after study participation. Chi-square tests were performed on the questionnaire answers, according to gender or education.

**Results:**

Of the 430 patients who returned the questionnaire, only 36.5% knew what a biobank was before participating in the study. Patients with less formal education were less informed by some sources (the Internet, newspapers, magazines, and our Institute). The final assessment test, taken after the multisource informed consent procedure, showed more than 95% correct answers. The information received was judged to be very or fairly understandable in almost all cases. More than 95% of patients were aware of participating in a biobank project, and gave helping cancer research (67.5%), moral obligation, and supporting cancer care as main reasons for their involvement.

**Conclusions:**

Our multisource informed consent information system allowed a high rate of understanding and awareness of study participation, even among less-educated participants, and could be an effective and easy-to-apply model for others to consider to contribute to a well-informed decision making process in several fields, from clinical practice to research.

Further studies are needed to explore the effects on the study comprehension by each source of information, and by other sources suggested by participants in the questionnaire.

## Background

Most recent studies aimed at improving patients’ understanding of the research or medical treatment in which they are involved concern comprehension of informed consent [1], rather than the participants’ awareness, motivation, or willingness [2]. These latter topics are little studied at present. Although patients’ involvement should be conscious and free from pressure or conditioning, the information phase is usually limited to the patient signing an informed consent form. However, a patient’s informed consent should also imply that the patient has received complete information and fully understands its meaning, so that conscious decisions can be taken [3-5].

However, when an information can be considered “complete”? No strong consensus exists as to adequate type and amount of information patients require, especially in the context of clinical research [6]. Signed informed consent forms can merely be considered as documentary evidence that patients have consented to participate, and had received the required information—but not that they thoroughly understood the given information.

The ethically valid process of informed consent includes indeed five elements: voluntarism, capacity, disclosure, understanding, and decision [6]. A review by Falagas and colleagues on informed consent process in clinical research showed that, in half of 30 considered studies, participants did not clearly understand the aim of clinical trials, or the fact that participation was voluntary, and that they could withdraw at any time. They were also unaware of the risks and benefits of treatment and the process of randomization. Moreover, sufficient understanding seems not to be directly attributable to the type of information provided but rather to the use of plain oral and written language, and to the time practitioners dedicated to clarifying any misunderstandings [7].

These topics raise nowadays an increased interest also in the field of biobanks [8]. Differently from what is sometimes thought, interventions to promote understanding of the aims and methods of biobanks, and enhanced awareness of their research applications, reportedly enhances individual willingness to participate in biobank collection [8-10]. Biobanks can be defined as “non-profit service facilities aimed at the collection and storage of human biological material to be used for diagnosis, biodiversity studies, and research” [11]. Cancer biobanks are of particular interest to both academic and industrial investigators; and the availability of large numbers of well-prepared samples is representative of the diversity and heterogeneity observed in tumour biology [12]. Without the contribution of patients, who provide their samples and associated information (therapies and outcome), improvement of treatments for cancer and other diseases could be severely delayed. Despite the great importance of patients’ connection with biobanks, they are often not aware of their fundamental role in medical research advance [13].

As with research projects and medical treatments, participants in biobanks should be informed about the purpose of their intervention, ways of execution, and related benefits and risks [14]. Unfortunately, often this is not the case: in one study, *none* of the participants was aware that he/she had samples stored in a DNA bank [15].

In 2006, the Centro di Riferimento Oncologico (CRO), National Cancer Institute, Aviano, Italy, established a biobank (CRO-Biobank) for cancer research purposes. It is a long-term source of human biological samples and associated information, collected at diagnosis and at consecutive therapeutic stages, in compliance with quality standards. Because the sole informed consent form, accompanied by technical information, was often unclear to patients [16,17], even in a simplified version [1], we implemented a multisource information procedure. The main purpose of this study was to evaluate the effectiveness of this approach through the use of a self-administered questionnaire. We compared patients’ knowledge before and after receiving information, and investigated the effect of the information sources: enhanced informed consent form, physician, biobank nurse or biologist, brochures, posters, the internet, television, etc. To find out how to enhance involvement and awareness, we also explored how patients expressed the need for further information, the sources they preferred, and the motivations that led them to contribute to the research. The questionnaire also investigated patients’ perception of the treatment of their individual and privacy rights to understand whether they felt respected as research stakeholders, and to reinforce their awareness and trust in the CRO-Biobank.

## Methods

From October 2009 to July 2011, 550 patients who had been admitted to the Centro di Riferimento Oncologico, IRCCS Aviano (CRO), National Cancer Institute, in North–East of Italy agreed to contribute to its biobank (CRO-Biobank) and were consecutively enrolled; 430 participants (78.2% of total; 86 men and 344 women; median age 56, age range 18–84) returned the filled-in questionnaire. Sample size was determined by number of cases presented during the study period. Eligibility criteria were: age ≥ 18 years, Italian speaking, and having histologically confirmed cancers or precancerous lesions. Patients’ main reason for non-participation was being too debilitated to fill out the form on hospital discharge.

**Figure 1:**
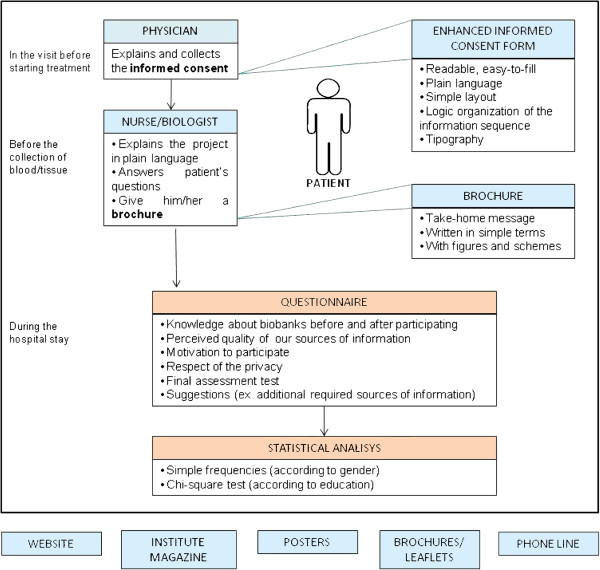
**Schematization of the multisource informed consent assessment procedure performed by the CRO-Biobank.** Sources of information are shown in light blue; assessment tools are shown in orange. Main panel: Informed consent procedure undergone by patients. Lower panel: Other information sources provided by the Institute.

The Institutional Ethics Committee (Comitato Etico Indipendente, Centro di Riferimento Oncologico, IRCCS, Aviano, Italy) approved the CRO-Biobank project. All patients provided written informed consent to participate. Participation involved collection of blood and, in case of surgery, tissue samples donated to the biobank for cancer research purposes. The CRO-Biobank designed a multiple-source patient information system (Figure 1). First, an enhanced informed consent form was designed as a readable, easy-to-fill model with a special attention to language, layout, organization of the information sequence, and typography. In the visit before starting treatment, physicians explained the contents of the form to patients, and collected the informed consent after giving all details of the CRO biobank purposes and the implications of the enrolment. Later, before collection of biological material, a biobank nurse (or a biobank biologist) gave further information in plain language, and answered any questions that might have arisen. Information included details on the purpose and activity of the biobank, on the informed consent form (such as the right to withdraw), and the existence of a dedicated phone line. The interview was performed contextually with the collection of anamnestic data and required about 15 minutes. Patients were also each given a brochure with a take-home message, written in simple terms, and containing figures and schemes [see Additional file 1]. The informational material can be found in various places around the Institute (i.e., brochures, posters, and the Institute magazine, available in display cases or stands), on the institutional website (http://www.cro.sanita.fvg.it/ricerca/set_biobanca.htm), and through a dedicated phone line.

During their hospital stay (for surgery, chemotherapy, or stem cell autologous transplantation) participants were each asked to take a written, self-administered questionnaire to assess their information levels and needs, aimed at investigating the effectiveness of our information system. The time elapsed between consent and response to the questionnaire was between 5 and 30 days. The questionnaire investigated patients’ knowledge of biobanks before and after participating in the study, the perceived quality of our informational methods, and how well the patients felt their privacy had been respected. It also included some demographic data and a final assessment test to measure the understanding of the form [see Additional file 2]. The questionnaire was validated prior to administration by administering it to a sample of 20 subjects and evaluating its understanding, validity and reproducibility. A few days later, we interviewed those subjects asking them the same questions to see if the answers were comparable to those marked in the questionnaire, and inquiring whether they had any difficulties in filling in answers.

Demographic characteristics (age, gender, education, and marital status), in addition to the admission ward, were collected to assess the study population. Age was divided into three groups: ≤ 50, 51–60, and ≥ 60 years. Participants were initially grouped according to gender; simple frequencies of demographic data were then assessed.

Patients were later divided into three groups by education level (those with elementary school, middle school, or high school/university education) to see whether formal education affected comprehension of information from different sources, as analyzed with a χ^2^test. Three participants were excluded from these analyses because of missing education data. Statistical analyses were performed using SAS software, version 9.1 (SAS Institute, Inc., Cary, NC).

## Results

Table 1 shows the distribution of demographical characteristics of participants according to gender. The study had more female patients than male patients, prevalently because of the higher contribution of the Gynaecology Oncology Surgery Unit. Nevertheless, no differences between variables were observed except for the Oncology Unit ward.

**Table 1:** **Distribution of personal characteristics of the 430 participants**, **divided by gender**; **2009**–**2011**

	**Gender**			
**Characteristics**	**Male (n = 86)**	**Female (n = 344)**	**Total**^**a **^**(n =430)**	***P *value**
	**N (%)**	**N (%)**	**N (%)**	
**Nationality**				
**Italian**	81 (94.2)	331 (96.2)	412 (95.8)	
**Non-Italian**	5 (5.8)	13 (3.8)	18 (4.2)	0.40
**Age (years)**				
**≤ 50**	25 (29.4)	123 (35.8)	148 (34.5)	
**51-60**	21 (24.7)	110 (32.0)	131 (30.5)	
**60**	39 (45.9)	111 (32.3)	150 (35.0)	0.06
**Marital status**				
**Single**	8 (9.3)	47 (13.7)	55 (12.9)	
**Married/cohabiting**	67 (77.9)	247 (72.2)	314 (73.4)	
**Separated/divorced/widowed**	11 (12.8)	48 (14.0)	59 (13.8)	0.49
**Education**				
**Elementary school**	19 (22.1)	78 (22.9)	97 (22.7)	
**Middle school**	27 (31.4)	100 (29.3)	127 (29.7)	
**High school/University**	40 (46.5)	163 (47.8)	203 (47.5)	0.93
**Oncology unit**				
**General oncology—surgery**	59 (68.6)	57 (16.6)	116 (27.0)	
**Gynaecology oncology—surgery**	–	191 (55.5)	191 (44.4)	
**Medical oncology**	19 (22.1)	17 (4.9)	36 (8.4)	
**Other**	8 (9.3)	79 (23.0)	87 (20.2)	< 0.001

^a^Numbers do not add up to the total because of missing values.

Table 2 reports patients’ answers about their knowledge of biobanks before entering the study. About one third of patients (36.5%) were aware of the existence of biobanks. Of these patients, 25.8% had attended elementary school, 36.2% middle school, and 41.9% high school or college; 30.7% derived information mainly from CRO sources (website, posters, phone line, brochures, etc.), 21.3% by television, and 17.6% by newspapers or magazines. Three sources of information (the Internet, CRO, and newspapers/magazines) shows statistically significant differences between patients who attended elementary school (less informed by these sources) and more educated patients. Information found on television or obtained from other hospitals did not differ between groups.

**Table 2:** **Distribution of answers given by patients to the questionnaire investigating their knowledge about biobanks before study participation**, **according to education**; **2009**-**2011**

	**Education**				
	**Grammar school (n = 97)**	**Middle school (n = 127)**	**High school/university (n = 203)**	**Total**^**a**^	***P *value**
	**N (%)**	**N (%)**	**N (%)**	**N (%)**	
**Were you aware of the existence of biobanks?**					
**No**	72 (74.2)	81 (63.8)	118 (58.1)	271 (63.5)	
**Yes**	25 (25.8)	46 (36.2)	85 (41.9)	156 (36.5)	0.03
**What was the source of information?**					
**Television**					
**No**	80 (82.5)	97 (76.4)	159 (78.3)	336 (78.7)	
**Yes**	17 (17.5)	30 (23.6)	44 (21.7)	92 (21.3)	0.54
**Newspapers/magazines**					
**No**	91 (93.8)	104 (81.9)	157 (77.3)	352 (82.4)	
**Yes**	6 (6.2)	23 (18.1)	46 (22.7)	75 (17.6)	0.002
**Internet**					
**No**	96 (99.0)	120 (94.5)	183 (90.2)	399 (93.4)	
**Yes**	1 (1.0)	7 (5.5)	20 (9.9)	28 (6.6)	0.01
**CRO**^**b**^					
**No**	79 (81.4)	89 (70.1)	128 (63.1)	296 (69.3)	
**Yes**	18 (18.6)	38 (29.9)	75 (37.0)	131 (30.7)	0.005
**Other hospitals**					
**No**	93 (95.9)	125 (98.4)	189 (93.1)	407 (95.3)	
**Yes**	4 (4.1)	2 (1.57)	14 (6.9)	20 (4.7)	0.08

^a^ Numbers do not add up to the total because of missing values.

^b^ Centro di Riferimento Oncologico, IRCCS, Aviano.

Table 3 shows participants’ perception of the quality of information received by the CRO-Biobank during enrolment, according to education. Most patients, 93.4%, had read the brochure, 83.6% reported being informed by a physician, and 89.2% by a biobank nurse or biologist. The information was evaluated as “very understandable” by the majority of participants (brochure: 61.4%; nurse/biologist: 61.7%; physician: 50.5%). More highly educated participants rated the sources of information as significantly clearer than did less educated participants. Overall, 53.8% patients rated the enhanced informed consent form to be “fairly understandable”, 44.9% as “very understandable”, and 2.3% as “insufficiently understandable”. Also, 56.1% of participants wished to receive more information on biobanks; 20.1% wished to be informed by multiple sources, 18.5% by CRO, 11.2% by websites (mostly patients with high school or university educations), and 4.9% by their general practitioner (mostly less educated patients) (data not shown).

**Table 3:** **Distribution of answers given by 430 patients regarding their perception of quality of information about biobanks received during CRO**-**Biobank multisource information process**, **according to education**; **2009**-**2011**

	**Education**				
	**Grammar school (n = 97)**	**Middle school (n = 127)**	**High school/university (n = 203)**	**Total**^**a**^	***P *value**
	**N (%)**	**N (%)**	**N (%)**	**N (%)**	
**What was the source of information?**					
**Brochure**					
**No**	6 (6.4)	10 (8.3)	11 (5.6)	27 (6.6)	
**Yes**	88 (93.6)	110 (91.7)	186 (94.4)	384 (93.4)	0.63
***How would you rate it?***					
**Unclear**	2 (2.4)	–	5 (2.7)	7 (1.9)	
**Fairly clear**	46 (54.8)	31 (29.3)	62 (33.0)	139 (36.8)	
**Very clear**	36 (42.9)	75 (70.8)	121 (64.4)	232 (61.4)	< 0.001
**Physician**					
**No**	5 (12.5)	8 (12.7)	19 (20.7)	32 (16.4)	
**Yes**	35 (87.5)	55 (87.3)	73 (79.4)	163 (83.6)	0.32
***How would you rate the information received?***					
**Unclear**	5 (11.4)	–	5 (6.3)	10 (5.3)	
**Fairly clear**	23 (52.3)	25 (38.5)	35 (44.3)	83 (44.2)	
**Very clear**	16 (36.4)	40 (61.5)	39 (49.4)	95 (50.5)	0.03
**Nurse/Biologist**					
**No**	9 (17.3)	7 (9.7)	13 (9.0)	29 (10.8)	
**Yes**	43 (82.7)	65 (90.3)	131 (91.0)	239 (89.2)	0.24
***How would you rate the information received?***					
**Unclear**	–	–	8 (5.7)	8 (3.2)	
**Fairly clear**	22 (48.9)	24 (35.3)	42 (30.7)	89 (35.2)	
**Very clear**	23 (51.1)	44 (64.7)	89 (63.6)	156 (61.7)	0.03
**How would you rate the informed consent form?**					
**Insufficiently understandable**	2 (2.2)	1 (0.9)	6 (3.1)	9 (2.3)	
**Fairly understandable**	59 (64.8)	61 (52.1)	95 (49.5)	215 (53.8)	
**Very understandable**	30 (33.0)	55 (47.0)	91 (47.4)	176 (44.0)	0.10
**Would you like to receive additional information about the CRO**-**Biobank?**					
**No**	41 (46.6)	55 (48.7)	76 (39.8)	172 (43.9)	
**Yes**	47 (53.4)	58 (51.3)	115 (60.2)	220 (56.1)	0.27

^a^ Numbers do not add up to the total because of missing values.

Table 4 shows distribution of answers regarding participants’ knowledge of biobanks after enrolment, according to education. The large majority of patients (95.5%) were aware of their participation in the CRO-Biobank (as all participants donated biological samples to our biobank, the question: “Did you donate biological material to CRO-Biobank?” intended to investigate whether they were aware of their participation). We also performed a trend test confirming that more educated participants were more aware of their participation (*P* = 0.02). The questions on their knowledge of biobanks were answered correctly by more than 95% of patients, with no differences observed according to education.

**Table 4:** **Distribution of answers given by 430 patients to the questionnaire investigating their knowledge about biobanks after being enrolled in CRO**-**Biobank and undergoing its multisource informed consent process**, **according to education**; **2009**-**2011**

	**Education**				
	**Grammar school (n = 97)**	**Middle school (n = 127)**	**High school/university (n = 203)**	**Total**^**a**^	***P *value**
	**N (%)**	**N (%)**	**N (%)**	**N (%)**	
**Did you donate biological material to CRO-Biobank?**					
**No**	8 (8.5)	6 (4.8)	5 (2.5)	19 (4.6)	
**Yes**	86 (91.5)	119 (95.2)	194 (97.5)	399 (95.5)	0.07
**Do you know what is stored in a biobank?**					
**Biological material**	85 (96.6)	117 (100.0)	193 (99.5)	395 (99.0)	
**Clinical records/scientific books**	3 (3.4)	–	1 (0.5)	4 (1.0)	0.03
**Do you know the end use of the CRO-Biobank material?**					
**Cancer research and care**	92 (100.0)	118 (100.0)	199 (99.5)	409 (99.8)	
**Informative/economic purpose**	–	–	1 (0.5)	1 (0.2)	0.59

^a^ The sum does not add up to the total because of missing values.

The reasons for participating in the CRO-Biobank (open question) were grouped into three main categories: to help cancer research (67.5%), to support care/for moral obligation (17.6%), and other (14.0%) (data not shown).

Over 98% of patients answered that the interview settings ensured their privacy and that the collection of biological samples was respectfully conducted (data not shown).

## Discussion

Active involvement of patients in the health sector is growing [18,19]. Patient involvement affects procedures and decisions regarding health policy [20], treatment [21], research [18,22], health technology assessments [23], genetic testing [24], and scientific advisory processes [19]. Patient involvement is thought to make health systems more user-friendly and information more accessible. Patient empowerment also involves respecting their rights and voices [25]. For these reasons, informed consent considerations cannot be ignored. Informed consent represents a very fundamental tool to achieve these goals; development of an efficient procedure that respects these principles is critical.

As per law and ethics, in many countries, physicians must collect informed consent from each patient or patient’s representative (e.g. parent or guardian) before enrolment in a study or initiating treatment [3,4]. This rule also applies to biobanks, as the biological material they collect is for scientific research purposes. Although the informational phase usually results in patients merely signing the informed consent form, this stage should also involve truly educating patients so that they can make informed decisions [1]. Despite growing interest in patients’ understanding of research in which they are enrolled, participants’ awareness and attitudes towards biological material donated for research are not well-studied [2]. Moreover, failing to properly inform patients about the purposes of human tissue sampling and use has caused a reduction of public confidence in biobanks, which can only be recovered by a transparency policy [26]. Issues raised in this investigation bear on both scientific and ethical perspectives [27]; we aimed to improve understanding, awareness and involvement of CRO-Biobank participants through a multisource information approach.

As no standard methodology for evaluating the informed consent process exists [28], we developed a self-administered questionnaire to assess patients’ information and satisfaction, and a final assessment test on the biobank purposes.

A review by Flory et al. evaluated effects of different interventions to improve participants’ understanding of informed consent for research, including multimedia interventions, enhanced consent forms, extended discussion, and miscellaneous methods. The review showed that these information sources do not consistently improve participants’ understanding, especially for less-educated patients. It appears that the only effective source of information is a one-on-one session where a study team member spends extra time explaining the study to the participant [1]. Our multisource information procedure included this type of approach among others; we added the support of a biobank nurse or a biologist to the physician to provide more explanation in simple language, particularly for less educated patients [15]. Most participants of this study were satisfied with the quality of the information received during the enrolment, as they rated each CRO-Biobank source of information “very understandable” (brochure, physician, nurse/biologist) or “fairly understandable” (enhanced informed consent form), although 56.1% expressed the wish for further information. Education level was associated with understanding: more educated participants rated sources of information as more understandable. This is in accordance with other studies [1]. Less-educated participants might, nevertheless, be poorly skilled in filling in the questionnaire, which could lead to underestimation of their understanding of the information [1]. Another biobank set a multisource approach to communication based on newsletters, external advisory boards, and focus group discussions in order to provide on-going feedback [10]. Participants expressed their desire to be informed only about changes in protocols or informed consent procedures; this result emphasizes the need for constant communication on these topics to maintain participants’ understanding, confidence, and trust.

Our study gave us insight into patients’ knowledge of biobanks prior to their participation therein. Only 36.5% were aware of the existence of biobanks: this percentage dropped to 25.8% in less-educated patients. Most participants who knew of biobanks before entering the study had found information through our Institute, which led to an overestimation of the percentage. Indeed, our goal of improving patients’ knowledge is not limited to the present study but is part of our biobank policy. The informational material can be found in various places around the Institute (i.e., display cases, stands), on-line, and through a dedicated phone line. The results of the study indicate that our efforts were successful for 30.7% of our patients (compared to 4.7% who answered that they had been informed by other hospitals). The information provided by our Institute was more effective than television (21.3%), which is the main source of information for less educated patients, and newspapers/magazines (17.6%), or the Internet (6.6%).

The most noteworthy result was that, with our multisource information approach, more than 99% of participants correctly answered questions about the purposes of biobanks. Moreover, our results showed that, unlike most studies, our approach allowed the information to be widely understood by less educated participants [1]. A survey among participants in another biobank, like ours, indicated that participants were motivated by a “pragmatic attitude” to contribute to research advancements; knowing what prompts individuals to participate helps define how to strengthen their involvement and awareness.

A novel aspect of this study is to investigate how patients preferred to be informed. Most preferred multiple sources of information. More educated patients preferred a website, while less educated patients opted for having information provided by their general practitioner.

The study showed wide awareness among participants; 95.5% of patients were aware of their involvement in a biobank (as all participants donated biological samples to our biobank, the question: “Did you donate biological material to CRO-Biobank?” was meant to see whether they were aware of their participation). These data are in accordance with studies by Toccaceli et al. (89.9%) [2] and Nakayama et al. (92%) [29], but in contrast to those of Moutel et al. (0%) [15].

The protection of personal rights in biobanks is a topic of great interest [30,31], and studies of public perception of biobanks have increased [30,32,33]. In contrast, we investigated the participants’ perceptions of this topic, rather than public perception. More than 98% of patients felt respected in their privacy and desires, and most rated the enhanced informed consent forms as “fairly understandable” to “very understandable”; these results represent an important aspect of the quality of our system and our intention to make patients feel part of the research.

Cancer patients face their diseases in personal ways; their views on participating in research or biobank projects vary, and can be different from the general population. For this reason, we directly investigated their understanding and awareness rather than perform a general population survey. Such surveys among cancer patients remain scarce but some articles have been published over the years [34-39]. In particular, Mancini et al. assessed cancer patients’ understanding about a biobanking informed consent process in the framework of a routine “opt-in” scenario [37]. Their results showed that 61.5% of patients who remembered giving consent and 31.5% of patients who declared not to have given consent, actually had given it. Moreover 41.3% of patients understood that consent implied giving access to their medical data. Scarce consensus exist about cancer patients’ reasons for participating in a biobank. In our experience, patients mainly contribute to aid cancer research (in accordance with the survey of Huber et al.) [39] and secondarily, to support care or for moral obligation; in other studies they contribute hoping for personal benefit [38,39], because of societal welfare or as an act of benevolence [38].

Nowadays, the need to collect informed consent for biobanking is somewhat controversial. Some authors proposed “opt-out” [40] or “opt-out-plus” procedures [36]. Whereas participants in opt-in procedures explicitly express their consent, in opt-out procedures, inaction signifies consent, and participants must affirmatively decline to take part, either orally or in writing. In some countries, such as Denmark, France and Belgium, this option is considered a sufficient consent modality for the use of residual tissue for research [40]. The surveys by Vermeulen et al. of cancer patients support adoption of opt-plus procedure, in which patients are informed about the possibility to opt-out, both verbally and by means of a leaflet [35,36]. In contrast, when Mancini et al. questioned cancer patients about their attitudes concerning informed consent, most felt that biobank research should require patients’ signed consent. Opting-in is the method suggested for including people in clinical research [41]; in most countries, it is the only allowed solution.

Regardless of the type of procedure that is chosen, the concept of informed consent is based on respect for participants’ autonomy and their right to control their medical care and research participation. Our system fulfils this condition from both ethical and legal points of view.

Although our informed consent procedure was designed and developed in an institutional biobank, its features can be easily implemented at low cost by other institutions thanks to its simplicity, which represents its major strength. Moreover it can be applied in several fields, from clinical practice to research, to improve patients’ awareness and understanding of information relevant to the informed consent process. Resources needed to apply this system are the following: (a) an initial investment of time to enhance the informed consent form (see Figure 1) and to format the graphic layout of the patient’s information sheet to create a brochure, a poster, and a web page; (b) a meeting to train staff about the project; and (c) involvement of a trained biologist/nurse who can give participants further information in plain language, and answer any of their questions, before the collection of samples site or through a phone line (estimated time ≤ 15 minutes for each participant). The last is the only resource that could lead to sizeable financial outlay— the cost of a nurse’s services if one is not already available. For our program, a research nurse (whose job was to collect samples and interview patients for our biobank) was trained with no additional cost to our institution.

Among the limitations of the study, we must consider potential bias due to participants who did not return the questionnaire, or who refused to answer some questions. Although we do not have the detailed demographical data regarding non-responders, the percentage of them was approximately the same distributed by department, pathology, sex and age. We assessed patients’ perception of the quality of each source of information, but this parameter does not indicate the real quality of the sources. As a starting point for future investigations of this topic, we suggest developing an assessment test to be given after having informed patients through each source. Further studies are also needed to determine whether, after a longer period (e.g. one year), participants still retain the disclosed information. The multisource information approach could also be optimized, for example by verifying which sources are the most efficient and excluding the others. To enhance the understanding of less educated participants, the results of our study suggest involving their general practitioners as a new source of information, as this was frequently requested by patients in the questionnaire.

## Conclusions

Our results showed information given to participants was well understood, that almost all patients acknowledged their awareness of their participation in CRO-Biobank, and that they were highly motivated, which implies that providing a well-informed consent process improves patient autonomy in conscious decision making. Our multisource information approach could be an effective and easy-to-apply model for others to consider in improving participants’ awareness and understanding of information provided in the informed consent process. It could also be a low-cost system if it is possible to involve a nurse or a biologist already working in the Institution.

## Competing interests

The authors declare that they have no competing interests.

## Authors’ contributions

SC participated in the design and coordination of the study, in the interpretation of data, and drafted the manuscript; JR was a trained participant in the informed consent process and aided in the collection of data; RT performed statistical analysis; TP performed diagnostic analysis for patients’ inclusion in the study; PDP conceived of the study; AS and VC participated in study design and coordination. All authors read and approved the final manuscript.

## Pre-publication history

The pre-publication history for this paper can be accessed here:

http://www.biomedcentral.com/1472-6939/14/30/prepub

## Supplementary Material

Additional file 1 **“CRO-Biobank brochure for patients”.** Informative brochure to be given to all participants. It explains in simple terms, with figures and schemes, the purpose and methods of CRO-Biobank, and implications of participating. Click here for file

Additional file 2 **“CRO-Biobank questionnaire for patients”.** Questionnaire designed to assess patients information levels and needs about biobanks before and after participating in the study. It aims at investigating the effectiveness of our information system. Click here for file
