# Supplementary material for: An effective multisource informed consent procedure for research and clinical practice: an observational study of patient understanding and awareness of their roles as research stakeholders in a cancer biobank
Source: BMC Med Ethics. 2013 Jul 30;14:30. doi: 10.1186/1472-6939-14-30 (PMC3734192; doi:10.1186/1472-6939-14-30)
Supplement: Additional file 1 — “CRO-Biobank brochure for patients”. Informative brochure to be given to all participants. It explains in simple terms, with figures and schemes, the purpose and methods of CRO-Biobank, and implications of participating. [file 1472-6939-14-30-S1.pdf]

CRO-BIOBANK

The CRO-Biobank (Aviano) was founded in 2006 to collect and preserve biological samples (for example, blood and tissues) fundamental for cancer research purposes.
The availability of a large number of organized samples helps research because each sample can be used in many different projects by diverse scientific groups. Ordinary citizens can facilitate the collection which would otherwise take many years by simply giving their consent to donate their biological material (from the collection of blood or other biological fluids, biopsies and surgical interventions).
In this way, all citizens can become promoters of scientific research.

The procedure is very easy:

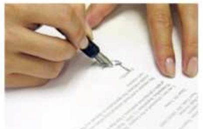

1. Signed informed consent (the opportunity to ask questions to trained health staff)

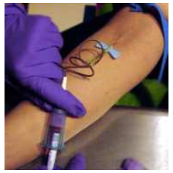

2. Blood (or other bio-logical material) collection

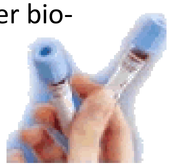

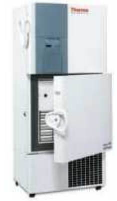

3. Freezing at very low temperatures (– 80℃ o – 196℃)

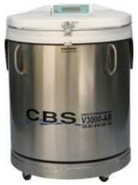

AIMS OF THE RESEARCH

Samples collected by the CRO-Biobank will be used for scientific research to help cancer prevention, diagnosis and therapy.
This type of research allows to:

- Increase the knowledge about different cancer types,
- identify the factors that can promote their development,
- Choose a more targeted therapy based not only on the type and stage of the disease, but also on the patient's characteristics.

For example, thanks to these studies, today there are new therapies which are no longer based on the "classic" chemotherapy, but on molecules that specifically attack only cancer cells. In this way, side effects are reduced and the possibility of healing is increased.

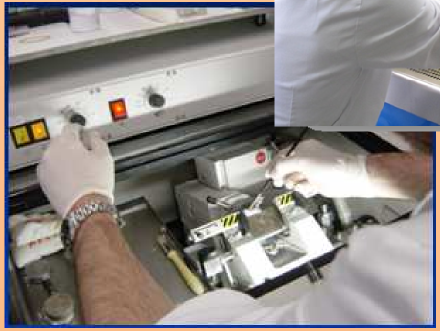

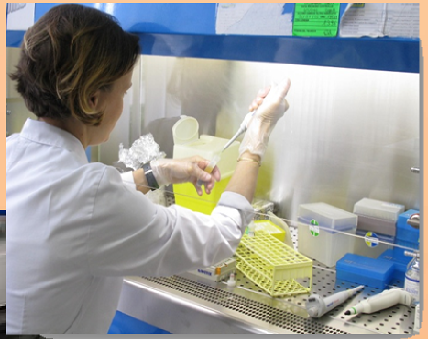

TECHNOLOGY

The management of our Biobank is well-advanced on the European scene by making use of innovative systems.
For example:

- the tubes used for the biological material preservation are marked with a special barcode which allows us to identify archived samples and easily recover them;

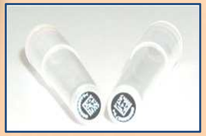

- the freezers (in which tubes are stored) equipped with wireless alarm systems that, in case of failure, warn the Biobank managers with a sms message;
- information relating to the health of the patient are updated thanks to computer programs that allow to maintain anonymity and respect, therefore, the privacy.
- the methods used for the collection, processing and storage of biological material in the Biobank ensure that any samples remain stable for many years and also available for future research.

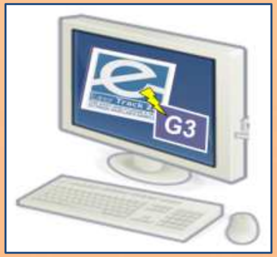

This project will quickly lead to a large collection of different biological samples from both healthy and sick people, which will be a valuable resource for research on different types of cancer. We strongly believe in this project and its potential, and we believe that the process of awareness that we are promoting will soon bear positive results. Every donation is a small drop which, with the commitment of all, will give a lot of new and advantageous information to improve the diagnosis and treatment of cancer.

STORAGE ROOM

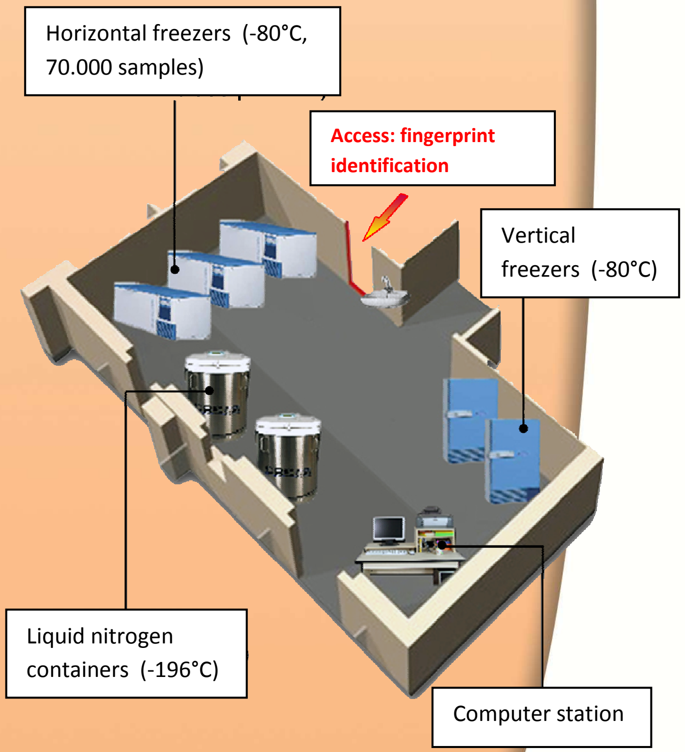

5 PER MILLE AL CRO

Grazie
per il tuo 5 per mille
a sostegno della
ricerca che cura

C.F. 00623340932

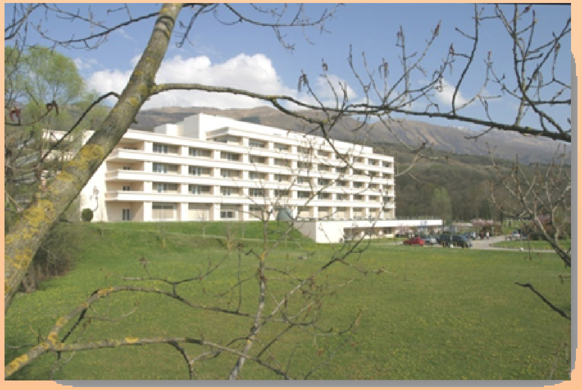

CONTACTS

For information or questions call:
Referent: 0434/659711 (Dr. Silvia Cervo)
Biobank: 0434/659571
0434/659292

NATIONAL CANCER INSTITUTE
CRO – Centro di Riferimento Oncologico

Via F. Gallini, 2 - 33081 Aviano (PN)
tel. +39 0434 659111
fax +39 0434 652182
P.IVA / C.F. 00623340932

LA BIOBANCA DEL CRO

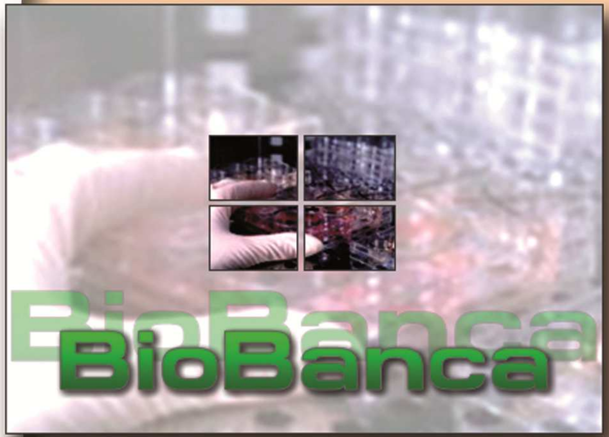

**Director:**	**Dr. Paolo de Paoli**
**Managers:**	**Dr. Vincenzo Canzonieri**<br>**Dr. Agostino Steffan**
**Referent:**	**Dr. Silvia Cervo**
**Tissue section:**	**Dr. Tiziana Perin**<br>**CPSE TSLB Ms. Barbara Canal**
