# Supplementary material for: An effective multisource informed consent procedure for research and clinical practice: an observational study of patient understanding and awareness of their roles as research stakeholders in a cancer biobank
Source: BMC Med Ethics. 2013 Jul 30;14:30. doi: 10.1186/1472-6939-14-30 (PMC3734192; doi:10.1186/1472-6939-14-30)
Supplement: Additional file 2 — “CRO-Biobank questionnaire for patients”. Questionnaire designed to assess patients information levels and needs about biobanks before and after participating in the study. It aims at investigating the effectiveness of our information system. [file 1472-6939-14-30-S2.pdf]

CRO AVIANO	Centro di Riferimento Oncologico<br>National Cancer Institute<br><br>CRO-BIOBANK	**President**<br>Dr. Paolo De Paoli<br>**Managers**<br>Dr. Vincenzo Canzonieri<br>Dr. Agostino Steffan

COGNITIVE QUESTIONNAIRE ON CRO-BIOBANK

Dear Sir or Madam, since we requested your contribution to the CRO-BIOBANK, regardless of whether or not you have given us your consent, we kindly invite you to answer this questionnaire. The compilation will take only a few minutes; you can answer by marking with a cross (eg. ☒) the response that you consider most appropriate.
Thank you for your kind contribution.

1. What is your gender?

☐ Male ☐ Female

2. What is your Nationality? (Please specify) ………………………………………..

3. How old are you? (Please specify) |__|__|

4. What is your marital status?

☐ Single ☐ Married/cohabiting ☐ Separated/divorced ☐ Widowed

5. What is your educational level?

☐ Elementary school ☐ Middle School
☐ High school ☐ University

6. Did you donate biological material (blood, tissue, etc.) to CRO-BIOBANK?

☐ NO ☐ YES

7. Would you briefly describe why you donated or refused to donate biological material to CRO-BIOBANK? (Please specify)..

8. Were you aware of the existence of BIOBANKS before today?

☐ NO (Skip to the questions on the reverse side of this sheet) ☐ YES

<u>If YES</u>, what was the source of information? (multiple answers are allowed)

	NO	YES	If YES, rating		
Television	☐	☐	☐ Unclear	☐ Fairly clear	☐ Very clear
Newspapers/ magazines	☐	☐	☐ Unclear	☐ Fairly clear	☐ Very clear
Internet	☐	☐	☐ Unclear	☐ Fairly clear	☐ Very clear
CRO	☐	☐	☐ Unclear	☐ Fairly clear	☐ Very clear
CRO-brochure	☐	☐	☐ Unclear	☐ Fairly clear	☐ Very clear
CRO-poster	☐	☐	☐ Unclear	☐ Fairly clear	☐ Very clear
CRO news	☐	☐	☐ Unclear	☐ Fairly clear	☐ Very clear
Other hospitals	☐	☐	☐ Unclear	☐ Fairly clear	☐ Very clear

9. With this questionnaire, you have been handed a flyer, did you read it?

☐ NO ☐ YES

If YES, how would you rate it? ☐ Unclear ☐ Fairly clear ☐ Very clear

10. In this hospital, who gave you information about CRO-BIOBANK and how would you rate this information?

	NO	YES	If YES, rating		
Physician	☐	☐	☐ Unclear	☐ Fairly clear	☐ Very clear
Nurse/Biologist	☐	☐	☐ Unclear	☐ Fairly clear	☐ Very clear

11. Would you like to receive additional information about CRO-BIOBANK?

☐ NO ☐ YES

If YES, by which sources? (multiple answers are allowed)

☐ CRO health personnel ☐ CRO news

☐ My family doctor ☐ More detailed brochure

☐ Video clips in the waiting rooms at CRO ☐ Website

12. How would you rate the Informed consent form, explaining the use and preservation modalities of the biological material?

☐ Unclear ☐ Fairly clear ☐ Very clear

13. Do you think that the interview was conducted in a setting (ward, ambulatory) in respect of your privacy?

☐ NO ☐ YES

14. If you have donated samples to the CRO-BIOBANK, do you feel that the procedure was conducted in respect of your will/availability?

☐ NO ☐ YES

Below you will be asked some questions related to the purpose of biobanks (select only one answer for each question).

15. Do you know what is stored in a biobank?:

☐ Clinical records ☐ Blood and other biological material ☐ Scientific books

16. Do you know the end use of CRO-BIOBANK material?

☐ Cancer research and care ☐ Informative purpose ☐ Economic purpose

17. Do you think that biobanks are useful to the health of the community?

☐ NO ☐ YES

****

Thank you for taking the time to fill out this questionnaire and for your contribution to the progress of the CRO-BIOBANK.
To ensure the anonymity of your responses, close the questionnaire in the white envelope that was given to you and deliver it to the Department or the ambulatory nurse.
